# Modeling screening, prevention, and delaying of Alzheimer's disease: an early-stage decision analytic model

**DOI:** 10.1186/1472-6947-10-24

**Published:** 2010-04-30

**Authors:** Nicolas M Furiak, Robert W Klein, Kristin Kahle-Wrobleski, Eric R Siemers, Eric Sarpong, Timothy M Klein

**Affiliations:** 1 Medical Decision Modeling Inc., Indianapolis, IN., USA; 2 Eli Lilly and Company, Indianapolis, IN., USA

## Abstract

**Background:**

Alzheimer's Disease (AD) affects a growing proportion of the population each year. Novel therapies on the horizon may slow the progress of AD symptoms and avoid cases altogether. Initiating treatment for the underlying pathology of AD would ideally be based on biomarker screening tools identifying pre-symptomatic individuals. Early-stage modeling provides estimates of potential outcomes and informs policy development.

**Methods:**

A time-to-event (TTE) simulation provided estimates of screening asymptomatic patients in the general population age ≥55 and treatment impact on the number of patients reaching AD. Patients were followed from AD screen until all-cause death. Baseline sensitivity and specificity were 0.87 and 0.78, with treatment on positive screen. Treatment slowed progression by 50%. Events were scheduled using literature-based age-dependent incidences of AD and death.

**Results:**

The base case results indicated increased AD free years (AD-FYs) through delays in onset and a reduction of 20 AD cases per 1000 screened individuals. Patients completely avoiding AD accounted for 61% of the incremental AD-FYs gained. Total years of treatment per 1000 screened patients was 2,611. The number-needed-to-screen was 51 and the number-needed-to-treat was 12 to avoid one case of AD. One-way sensitivity analysis indicated that duration of screening sensitivity and rescreen interval impact AD-FYs the most. A two-way sensitivity analysis found that for a test with an extended duration of sensitivity (15 years) the number of AD cases avoided was 6,000-7,000 cases for a test with higher sensitivity and specificity (0.90,0.90).

**Conclusions:**

This study yielded valuable parameter range estimates at an early stage in the study of screening for AD. Analysis identified duration of screening sensitivity as a key variable that may be unavailable from clinical trials.

## Background

Alzheimer's Disease (AD) is a well-known, degenerative neurological disorder primarily affecting the elderly with the potential for extremely poor quality of life and high treatment costs. The increasing age of the general population and the incidence of AD in the elderly means that the burden of AD will continue to increase, with a projected prevalence of 8 million by the year 2050 [1,2]. Current symptomatic treatments have a modest benefit on cognitive symptoms of AD, but there is little evidence that they modify the underlying cause of AD, thus limiting their use as a preventive treatment in asymptomatic patients.

In parallel to current drug development efforts aimed at slowing the underlying progression of AD, various biomarkers are currently being researched as diagnostic tools. These biomarkers include a variety of imaging techniques as well as assays that evaluate blood and cerebrospinal fluid levels of markers that are believed to be AD-specific [3-8]. These markers have demonstrated modest predictive value for identifying patients at risk for future conversion to AD when studied in patients who already have mild cognitive defects [5-8]. Specifically, the sensitivity of this test for conversion to AD is 0.87 and the specificity is 0.78, representing the current state of knowledge in AD screening.

What is lacking in this parallel drug and biomarker development is a framework for understanding how these efforts might impact the onset of new cases or delay of symptom onset. Researchers commonly use computer simulation models to compare newly-developed treatments or screening tools to existing standards of care [9-14]. Modeling techniques are helpful to payer authorities and policy makers who consider cost-effectiveness of interventions when prioritizing research funding and making coverage and reimbursement decisions. These same modeling techniques can also be useful tools for clinicians and researchers to establish the appropriate population to treat or narrow the efficacy range needed for a drug in early stages of development to be commercially viable. The application of modeling techniques to biomarkers or imaging techniques may help address questions related to the value of testing. Questions include:1) What risk level justifies screening?, 2) At what age is it optimal to recommend that patients be screened?; 3) When does screening become unnecessary given a series of negative tests? In addition, it is critical to understand the potential benefits of re-screening within the expected age-range of disease onset.

Computer simulation techniques can be used to answer the above questions by synthesizing the properties of the screening test itself, the disease process, and potential therapies for treating and preventing the disease. Quantifying sensitivity and specificity and understanding their predictive value is the primary objective in the early stages of development of screening and treatment. Only when sensitivity and specificity are linked to effectiveness of a potential prevention therapy do the benefits to patients and society from screening become apparent. The field of AD research is at a critical juncture to benefit from modeling: biomarker-based screening measures are under development as are therapies that may modify the underlying neuropathological disease process.

The objective of this study is to synthesize current knowledge of the best-understood parameters and major components of AD-screening: AD biomarkers, screening strategies, prevention therapy, and progression of AD upon diagnosis to quantify patient-specific outcomes over a wide range of hypothetical scenarios. Collecting the variables and quantifying potential outcomes at an early stage provides a foundation for decision and policy makers to approach the issues of screening for AD *a priori *in contrast to the historical approaches to other disease state screening.

## Methods

This being one of the first simulation models evaluating screening and treatment to delay or prevent clinical AD, we provide explicit assumptions related to the base-case population and their adherence and persistence with treatment when a screen is positive. This allows an analysis that focuses on the impact of the attributes of the screening test on generalized AD outcomes. The assumptions characterizing the population to screen, rescreening, and persistence are examined through sensitivity analysis; therapy adherence, emotional consequences of screening, and adverse events of future treatments are addressed in the discussion and limitations sections. Of primary significance to this model is that the presence of Alzheimer's disease neuropathology identified on screening would precede the onset of clinically significant symptoms that would be diagnosed as dementia of the Alzheimer's type. Therefore, this is a true screening model for the presence of disease. For simplicity, we use "AD" to refer to clinically-diagnosable dementia. A positive screen means that there is presence of AD neuropathology but not necessarily clinical AD.

### Decision Analytic Model

The screening model is programmed as a deployed software application programmed in the CafeSim™ Java Simulation Development Environment. This tool is designed for the development of decision models ranging from decision trees, Markov models, Monte Carlo microsimulations (fixed-time advance or time-to-event), to discrete event simulation with queuing. Applying time-to-event (TTE) to screening for AD was undertaken to provide maximum flexibility for analysis on the attributes of screening tests and treatments that postpone disease onset.

**Figure 1:**
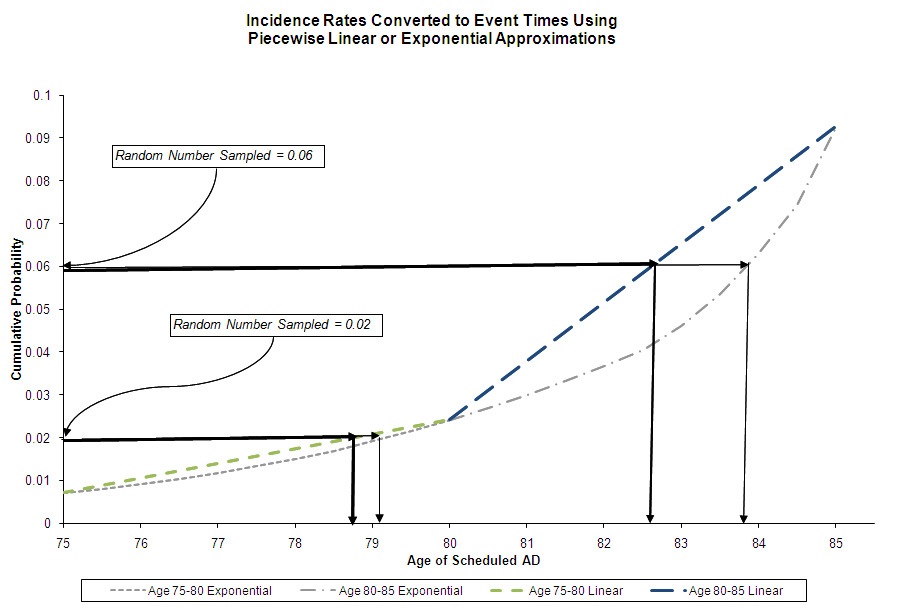
**Incidence rates converted to event times using piecewise linear or exponential approximations**. Example of a female screened before age 70, with incidence of AD at age 77 = 0.003, at age 82 = 0.074, at age 85 = 0.03. The y-axis is the cumulative probability of AD before the time shown.

**Figure 2:**
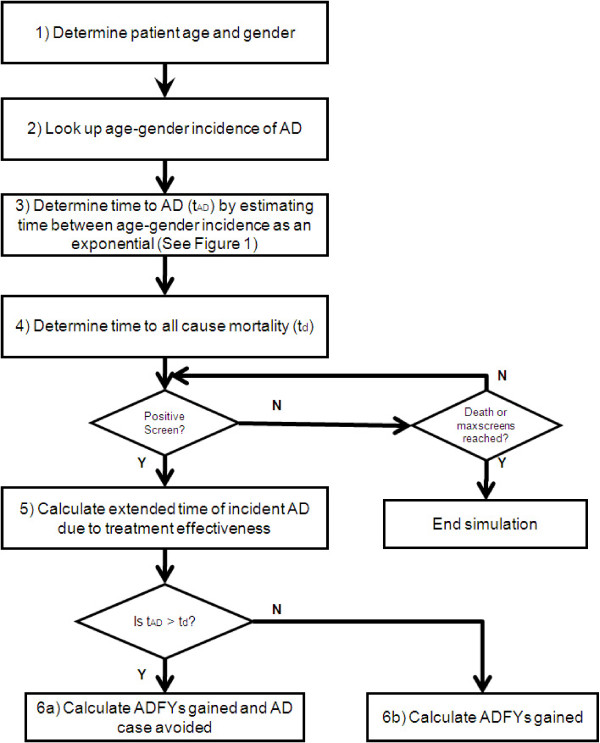
**AD screening model algorithm**.

The TTE distribution for an AD diagnosis has been constructed using incidence data from the literature [15,16] in conjunction with U.S. census data [17]. In the same manner as a recent model of colon cancer screening [18], the model assumes that AD onset can be represented by a non-homogeneous Poisson process [19,20]. Figure 1 shows two approximations (linear and exponential) of the cumulative hazard curve of females between ages 75 and 85. For each simulated patient a random number is drawn to determine if a hazard will occur and if so in what piece (age) of the distribution. Smoother approximations (e.g. using exponentials with piecewise linear hazards) between age strata provide more realistic estimates between adjacent strata. Figure 1 shows that at earlier ages when the hazard is low, the discrepancy between approximation methods is small. However, the exponential approach captures the phenomena whereby incidence changes substantially between adjacent pieces. Figure 2 summarizes the general flow of the AD model algorithm. Table 1 contains a complete list of literature based default inputs.

**Table 1:** Input Parameters for Base Case Model.

Screening					
Sensitivity of Screening Test (0-10 yrs)	0.87 [7]				

Sensitivity of Screening Test +10 yrs (= 1-specificity)	0.22 [7]				

Simulated Age at First Screening	70				

Interval Between Screens	Once				

**Treatment**					

Effectiveness	50%				

**Adverse Event Rate (Years After Initiation)**					

	**Years After Initiation**				

	0-1	1-5	5-10	10-15	15-20

AE Rate	10%	2%	2%	2%	2%

Discontinue Treatment Given AE	No				

AE Monitoring Interval (First Year)	0.5 yrs (= 2 in year 1)				

**General**					

% Cohort Male	46% [17]				

Annual Discount Rate	3%				

**Treatment Persistence (Discontinuation)**					

	In first 6 months			In second 6 months	

Alzheimer's Disease CI Model 1 [24]	0%			68%	

Alzheimer's Disease CI Model 2 [26]	0%			47%	

Breast Cancer Recurrence - Tamoxifen[21]	17%			5.1%	

Hypertension ACE Inhibitors [23]	25%			4%	

**Incidence Rates**					

	**Age Gender Probability Patient Will Develop AD **[15,16]				

	Male		Female		

50-54 (Value at age 52 in distribution)	0		0		

55-59 (57)	0		0		

60-64 (62)	0		0		

65-69 (67)	0.0007		0.0002		

70-74 (72)	0.0014		0.0004		

75-79 (77)	0.0025		0.003		

80-84 (82)	0.0065		0.0074		

85+ (Constant for ages 85-100)	0.016		0.03		

### Probabilities and Assumptions

Base-case model assumptions include: 1) The initially simulated cohort of patients are all screened; 2) No patient has AD at the time of screening; 3) All patients initiate prevention therapy upon positive screen; 4) Patients are 100% adherent to therapy (i.e., they take all doses as prescribed); 5) Similar to tamoxifen to prevent breast cancer recurrence, 22% discontinue treatment in the first year, but the rest persist until AD onset) [21]; 6) Patients are screened once at age 70; 7) The upper bound of the model timeframe is 100 years of age, so patients scheduled to get AD beyond age 100 do not get AD; 8) The effectiveness of treatment is not dependent on how far before disease onset it is given. The base-case model simulates screening at age 70 in keeping with the incidence of AD typically occurring later in life Screening as early as age 50 and late as age 80 are tested in sensitivity analysis.

The age-gender probabilities of AD diagnosis used to construct the TTE-AD distribution are based upon an analysis of patients from the Framingham study [15]. This study was chosen because it was a longitudinal, population-based, U.S. cohort that provided incidence and lifetime risk of AD adjusted for competing risk of death.

The effectiveness of AD prevention therapy is expressed as the percentage of time by which it would delay a patient's progression to AD. The default effectiveness in the screening model is 50%. Therefore, if a patient were scheduled to transition to mild AD in 2 years, then 2 years of prevention therapy would delay that transition by 1 year, extending the pre-mild AD period from 2 to 3 years. If the patient remained on therapy until onset the maximum delay would be 2 years (t/2+t/4+t/8 ...--> t).

Sensitivity (default; 0.87) and specificity (default; 0.78) are based upon a cerebrospinal fluid study measuring the ratio of Aβ-42 to Aβ-40. The cohort was followed-up to conversion from "mild cognitive impairment" (MCI) to AD for a mean of 5.2 years (range 4.0-6.8 years) [7].

It is rare that a screening test that does not detect a genetic marker has an infinite timeframe on sensitivity. More likely is that the time before onset at which a biomarker is detectable is a function of when the pathological process begins, implying that test sensitivity and specificity should be a function of time until onset. Given the lack of data on characteristics of this distribution our model uses a base-case assumption that there is a time limit on the sensitivity of a screening test to the biomarkers for AD and sensitivity analysis is conducted on this assumption. Although evidence from current clinical literature indicates a shorter mean follow-up time [7,8,22], clinical expert opinion suggests that currently recognized markers are legitimate within a range of 10-15 years. Consequently, we assume the base-case timeframe to be 10 years on sensitivity. If a patient is scheduled to get AD beyond the 10-year limit we assume that the rate of true positives is 1-specificity, i.e. using the false positive rate, assuming that patients more than 10 years from AD onset are indistinguishable from those who would never get AD. As discussed earlier, we have populated the base case model relying on simplifying assumptions to avoid erroneous conclusions about the screening test itself. In particular, we assume that once prevention treatment is initiated all patients are 100% adherent until mild AD diagnosis. This is an unlikely scenario in clinical practice but the adverse event profiles, onset of therapeutic effect, and cost of novel AD therapies such as gamma secretase inhibitors and antibodies are still unknown. However, we address adherence to therapy in the discussion and limitations sections. Therefore, we chose to evaluate the impact of persistence in sensitivity analysis by modeling persistence in a pattern similar to previous AD studies as well as prevention or treatment of other diseases found in the literature [21,23,24]. Specifically, we have modeled persistence in a manner similar to that for the prevention of breast cancer relapse in remitted patients taking tamoxifen [21]. We have also used persistence rates for studies in AD patients treated with cholinesterase inhibitors. These likely represent a lower bound for persistence due to their limited effectiveness and adverse event profile [25,26]. Finally, we have included analysis using data from literature on persistence with hypertension and statin therapies as an example of prevention therapy for insidious disease [23,27] as well as an examination of unending persistence.

### Outcomes of the Analysis

This model provides the following relevant outcomes:

1. AD-free years (AD-FYs)

2. Number of patients to AD

3. Mean years of AD prevention treatment

4. Number of patients recommended for treatment on first screen

5. Number Needed to Screen (NNS) to avoid one case of AD

6. Number needed to treat (NNT) to avoid one case of AD

7. AD-FYs gained per person year of prevention treatment

8. AD cases avoided per person year of treatment

One-way sensitivity analysis was performed by varying parameters within clinically relevant ranges. The results of these analyses are displayed as tornado diagrams. Each bar in the tornado diagrams represent the range of output values achieved varying one of the inputs listed below:

1. Treatment effectiveness

2. Patient age at initial screen

3. Re-screening interval

4. Years of screening sensitivity

5. Persistence patterns

**Figure 3:**
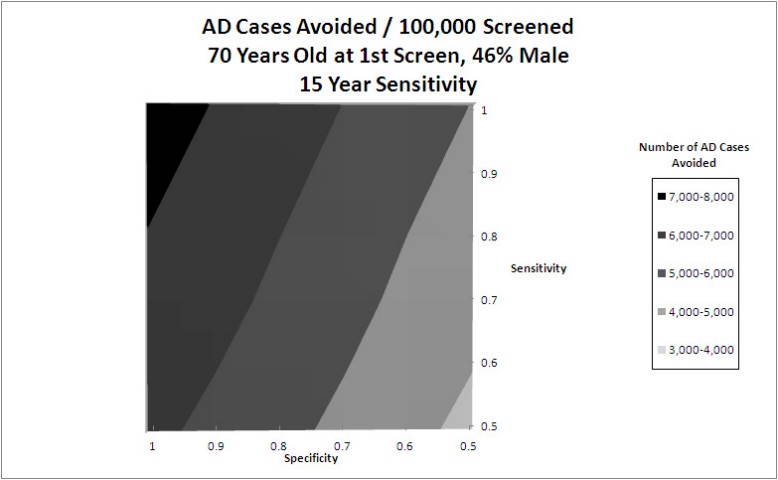
**Two-way sensitivity analysis of screening sensitivity and specificity on AD cases avoided**. AD cases avoided/100,000 screened, Age 70 at first Screen, 46% male, 15-year sensitivity.

The input with the greatest impact on the output in question is at the top of each tornado diagram and those with the least are at the bottom. Additional two-way sensitivity analysis on screening sensitivity and specificity was performed to determine their combined effect on the number of AD cases avoided in the general population (Figure 3).

## Results

Table 2 shows the base case results where the model predicted an increase in AD-FYs through delays in onset and a reduction of 20 AD cases per 1000 individuals screened. Auxiliary calculations showed that approximately 61% of the incremental AD free years gained were among the patients that avoided AD, the rest were gained by patients who had their AD onset delayed. The total number of years of treatment per 1000 screened patients was 2,611 reflecting the conservative assumption that screening is performed once at age 70 as well as the application of conservative persistence assumptions. Additionally, the NNS was 51 and NNT 12 to avoid one case of AD when screening 100,000 patients given default incidence.

**Table 2:** Base Case Results (Per 1000 Screened).

	No Screening	Screening	Incremental
Alzheimer's Disease Free Years	13,677	13,834	+ 157

Alzheimer's Disease Cases	113	93	- (20)

Total Years of Treatment	0	2,611	+ 2,611

Number Recommended for Tx	0	236	+ 236

			

NNS to Prevent 1 AD Case	51		

NNT to Prevent 1 AD Case	12		

AD-FY Gain/Person Yr of Treatment	0.0602		

AD Case Avoided/Person Yr of Treatment	0.0076		

Figure 3 displays a two-way sensitivity analysis of test sensitivity and specificity on AD cases avoided, when screening at age 70 and the duration of sensitivity is 15 years. The duration of sensitivity represents the period during which a marker for AD could be detected. Therefore, outside the timeframe of test sensitivity, 1-specificity is used for the sensitivity. Results indicate that a perfect test followed by perfect adherence to a 50% effective treatment could prevent AD in 7-8% of people screened (7,000-8,000 cases avoided per 100,000 persons screened). Nearly a 20% reduction in specificity or a 40% reduction in sensitivity is needed to reduce AD cases avoided by an absolute 1% (1000 per 100,000 persons screened). The reference case with a highly specific and sensitive test (0.90, 0.90) indicates screening has the potential to avoid AD in 6-7% of a screened population. For moderate rates of sensitivity and specificity (0.80, 0.80) the proportion avoiding AD is slightly below 6%. Other two-way analyses (not shown) indicated that for a screening test with shorter duration of sensitivity, test sensitivity and specificity were even less important.

**Figure 4:**
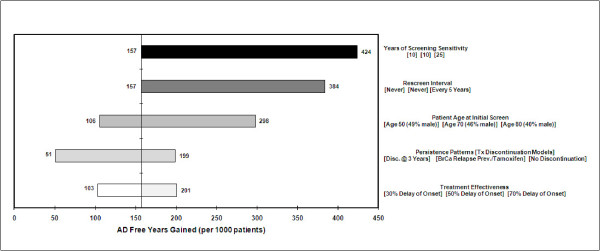
**One-way sensitivity on AD-Free Years (ADFYs)**.

**Figure 5:**
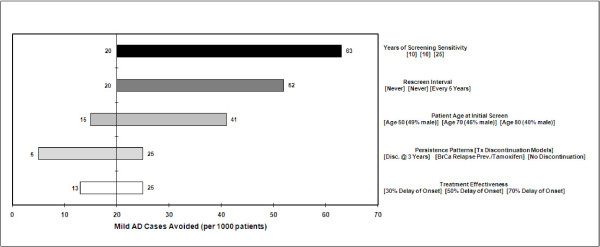
**One-way sensitivity on mild AD cases avoided**.

Results for one-way sensitivity analysis summarized in Figures 4 and 5 show the range of variation due to uncertainty in five additional inputs. In general, these results indicate that years of screening sensitivity and the rescreen interval are the two main drivers of AD-FYs (Figure 4) and AD cases avoided (Figure 5). The upper range of sensitivity duration for one-way analysis was 25 years and yielded 424 AD-FYs and 63 AD cases avoided. These may be considered extreme bounds as it is likely that screening sensitivity to detect a non-genetic marker would be no more than 10-15 years prior to onset. As expected, the frequency of rescreening improves AD-FY and AD cases avoided.

**Figure 6:**
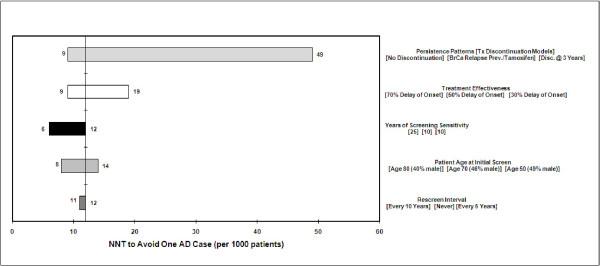
**One-way sensitivity on NNT**.

**Figure 7:**
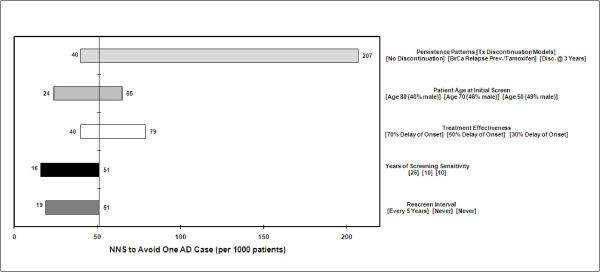
**One-way sensitivity on NNS**.

Additional one-way sensitivity analyses (Figures 6 and 7) showed that therapy persistence yielded the widest range of values for NNT and NNS outcomes, reflecting the underlying effect of treatment effectiveness. Given a discrete effectiveness (50% in the base case) the longer patients remain on therapy the greater the benefit and the fewer NNT and NNS required for a success. In the case of AD, this means that patients avoid the debilitating decline of AD and die from other causes.

## Discussion and Conclusions

To our knowledge, few studies in the literature have attempted to model screening for AD. Previously published studies have used a Markov modeling framework to evaluate the cost-effectiveness of treatment of AD (e.g. donepezil) [28-31]. First, these studies are limited in that they are treatment models for AD and do not contain screening components. Second, these studies rely on Markov cohort models which cannot represent the dynamic nature of cognitive impairment in patients of different ages and gender. Third, the inherent fixed-time advance mechanism may artificially limit the flexibility in evaluating screening options and may miss interim opportunities to intervene on behalf of patients. Finally, Markov models are cumbersome when expansion of health states, attributes, and assumptions are necessary. This model provides a framework within which key screening parameters can be tested, adjusted, and easily expanded as the role of AD screening takes shape.

Our TTE simulation establishes a foundation for numerous sensitivity analyses by simulating the many variables associated with AD and its dynamic nature. Additionally, there are potential implications of this model in terms of policy debate and establishing screening guidelines should future developments warrant them. As has been the case with mammograms [32], pap smears [33] and colorectal cancer screening [34,35], modeling can be a valuable tool in framing questions related to screening and defining a range of parameters within which society can benefit. Recently published results on screening for prostate cancer highlight a potential benefit from early stage modeling when determining the net benefits to patients and society [36]. Early, thoughtful, structured analyses akin to those presented in this work can potentially play a role in allaying societal stigmas associated with positive AD screens by assuring all stakeholders that intervention is beneficial.

In keeping with the study objective we have determined through hypothetical, credible scenarios that the effectiveness of screening for AD is most influenced by: Screening test sensitivity, duration of test sensitivity, efficacy of AD prevention therapy, persistence on treatment, and initial screening age. In particular, these analyses have allowed us to draw focus to the age to begin screening and possibly, the age at which to stop. For example, AD incidence is relatively low before age 80, but after 85 there may be little to be gained from delaying AD, due to death from other causes when only considering death and quality of the end of life. With a starting age of 70, increasing the number of years during which a test is sensitive rapidly improves the effectiveness of screening. If such an increase in time-frame of sensitivity is not possible, the model allows the user to define a range in which re-screening adds value.

We have chosen to limit the inputs, states, and assumptions in this version of the model to provide an uncluttered foundation for the fundamental variables involved and their basic interactions. Although this serves the purpose of creating a scalable instrument, it stands as a limitation in the short term. First, the narrow age-gender cohort we have chosen does not consider subpopulations. It is likely that if screening for AD were to become viable, it would be most effective and cost-effective in high-risk cohorts. This model does not currently attempt to address risk in subpopulations through attributes known to be indicators of a higher probability of AD later in life. Subpopulation analysis is likely to be the first expansion of the model in the future when questions on cost-effectiveness come to the fore.

Second, the model is limited by the lack of data on the precursor health states of mild AD. Indeed the diagnostic criteria and their corresponding accuracy are ambiguous within the current literature [37-39]. A consistent clinical approach to evaluating precursor states may encourage ongoing engagement from stakeholders and prevent more cases later in life. A lack of a definitive precursor state may lead to lower adherence and persistence to therapy as if it were not needed.

Third, the large amount of uncertainty in the AD screening test is the primary motivation for this study but it remains a limitation as well. We have attempted to adopt sensitivity and specificity assumptions that pass face validity according to the state of knowledge in AD research and the expectations of clinicians in the AD field. The assumptions in the model are based upon studies reporting conversion from MCI-like states to AD and may represent an overestimate of future screening test attributes. However, if a screening test exceeds expectations, is low-cost, and is relatively non-invasive, the screening sensitivity and specificity may be underestimated. Ultimately acceptable thresholds for screening characteristics incorporated into future clinical practice will surely address fundamental questions: Is society willing to accept a screening test less sensitive and specific compared to screening methods currently considered acceptable in the general public (due to the increased incidence of AD with an aging population)? Or, will the AD screening test be held to a higher standard due to patient anxiety and potential stigmatization on a positive screen for AD?

Fourth, there is no data to directly support persistence assumptions as the hypothetical therapies alluded to here are still in development. However, we have made an attempt to provide estimates using literature-based values for other well-known conditions spanning the spectrum from hypertension to breast cancer. These proxy states are the current best estimates until the efficacy and side effect profiles of novel AD therapies come to light.

Additionally, there are no data yet available to appropriately inform how the emotional consequences of screening for AD should be parameterized. The trade-off between the anxiety of screening and the long-term consequences of disease are difficult to quantify, except over very short timeframes when evaluating quality-of life adjustments. Moreover, recent developments in age thresholds and policies when screening for breast cancer indicate that the effect of anxiety in screening paradigms can shift. Avoiding undue anxiety when screening women at age 40 has been cited as a benefit to extending the initial screen age to 50, but some evidence suggests that patients understand the concept of false positives and are willing to tolerate periods of anxiety to prevent cases of death due to breast cancer [40,41]. Adherence to therapy and adverse event profiles, inextricably linked to the stigma of disease, represent possibly the most complex interaction of factors that might impact future AD screening efforts. This study does not attempt to address these phenomena but the simulation contains functionality to accept them at will should evidence arise to inform parameter estimates.

Finally, the duration of the sensitivity of the test remains a limitation. Ideally, the marker being screened for would have a long duration of sensitivity which would be encouraging for future use of the test. This is rarely the case (except in the case of a genetic marker) and the existing base model uses a 10-year duration of sensitivity that possesses face validity in the eye of clinicians experienced in the diagnosis and treatment of AD. Our assumption of 10 years of sensitivity has necessarily placed focus on age 70 as the most likely age of initial screening. Screening at younger ages increases the influence of the specificity of the screening test and screening at ages much later limits potential benefits and may be impractical due to the characteristics of therapy, specifically, adverse events. We have intentionally avoided cost estimates in this study given that the screening and therapy are hypothetical (i.e., no market value available). However, the age at initial screen may cover a wide range due to the high annual cost of AD cases to payers, caregivers, and society in general. Thus, screening at age 80 and delaying the onset of AD beyond the time within which other causes of death prevail might improve overall quality at the end of the patient's live (versus severe cases of AD, for example).

Despite these limitations, the model provides a novel, useful heuristic for researchers and clinicians as they design studies to aid further development in AD and other neurodegenerative diseases, such as Parkinson's disease. The TTE approach is well-suited to assessment of screening and intervention of neurodegenerative diseases because of the need to account for the high degree of variability in disease progression and potential differentiating risk factors. This study has met a critical object in that it has provided a range of estimates for outcomes that will be helpful in the early debate of the issues relating to screening and therapy for AD.

## Competing interests

N. Furiak, Medical Decision Modeling Inc., Employee; Eli Lilly and Company, Consultant; Medical Decision Modeling Inc., Stock Shareholder (directly purchased); R. Klein, Medical Decision Modeling, Inc., Employee; Eli Lilly and Company, Consultant; Medical Decision Modeling, Inc., Stock Shareholder (directly purchased); K. Kahle-Wrobleski, Eli Lilly and Company, Employee; E. Siemers, Eli Lilly and Company, Employee; T. Klein, Medical Decision Modeling, Inc., Employee.

## Authors' contributions

NMF researched and designed the model, assisted in programming, performed analysis, and wrote the manuscript; RWK assisted in model design and manuscript preparation, KKW helped design the model and prepare the manuscript; ERS consulted on the clinical aspects of the model, helped design the model, and helped edit the manuscript; ES helped design the model and prepare the manuscript; TMK programmed the model and performed analysis. All authors have read and approved the final manuscript.

## Pre-publication history

The pre-publication history for this paper can be accessed here:

http://www.biomedcentral.com/1472-6947/10/24/prepub
